# Why does research matter to psychiatrists?

**DOI:** 10.1177/23982128241305866

**Published:** 2025-01-11

**Authors:** Lindsey I Sinclair, Laura Ajram, Gertrude Seneviratne, Derek Tracy, Hugo Critchley

**Affiliations:** 1 Dementia Research Group, Bristol Medical School, University of Bristol, Bristol, UK; 2 Academic Faculty, Royal College of Psychiatrists, London, UK; 3 British Neuroscience Association, Bristol, UK; 4 South London and Maudsley NHS Foundation Trust, London, UK; 5 South London Partnership, London, UK; 6 Brunel University Medical School, London, UK; 7 Department of Psychosis Studies, Institute of Psychiatry, Psychology & Neuroscience, King’s College London, London, UK; 8 Department of Psychiatry, Imperial College London, London, UK; 9 Brighton and Sussex Medical School, University of Sussex, Brighton, UK

**Keywords:** Academic psychiatry, psychiatric research, psychiatric careers, reproducibility

At the 2024 Royal College of Psychiatrists International Congress, the British Neuroscience Association (BNA) helped to explore the theme of why research matters to psychiatrists. There is a common perception that research is something that happens elsewhere and that research skills are less important for full-time clinicians. The recovery trial (https://www.recoverytrial.net/) during the COVID-19 pandemic was a superb example of how all clinicians can contribute to research, with life-saving outcomes (Recovery Collaborative Group, 2021). Research is relevant to all medical specialities and, over the last century, has contributed to enormous changes in clinical practice and major improvements in clinical outcomes.

From the clinician’s point of view, becoming involved in research can increase job satisfaction, contribute to the development of a stimulating portfolio career and most importantly create more curious clinicians who feel more empowered and able to address clinical dilemmas that they encounter in their practice. From the National Health Services (NHS) Trust’s point of view, having research-active clinicians can have positive effects on patient outcomes (Ozdemir et al., 2015) as well as staff recruitment, retention and job satisfaction (Rees et al., 2019). We have also argued that having a more research-active workforce can contribute to a broader positive organisational culture of education, make services more efficient and contribute to effective national healthcare policy (Critchley et al., 2024).

Over the last decade, mirroring national trends in medicine more broadly, the number of academic psychiatrists in the United Kingdom has plummeted. Data from the Medical Schools Council 2023 survey (https://www.medschools.ac.uk/clinical-academic-survey) demonstrated a reduction of a third in full-time equivalent academic psychiatrists between 2004 and 2023, despite a major expansion during that period of the number of medical students. Does this decline matter? Psychiatrists in specific academic roles have had extensive specific training in research methods and usually hold an MD or PhD degree. This becomes relevant when thinking about the quality of research. The reproducibility crisis in research (Munafò et al., 2017) has taught us that getting study design right is critical, for example, regarding sample size and analysis techniques (de Vries et al., 2023). Increasingly, science is a collaborative endeavour (Grieve et al., 2023) and the study of psychiatric illness is using ever more complex neurobiological techniques in conjunction with broader epidemiological and psychosocial approaches. For example, the rabies virus has been used to map the circuits involved in parenting (Kohl, 2020; Kohl et al., 2018), and optogenetics is helping us to understand depression (Deisseroth, 2017; Ferenczi et al., 2016; Warden et al., 2012). Poor-quality research undermines our credibility as a community, as emphasised by the BNA’s campaign for Credibility in Neuroscience (https://www.bna.org.uk/about/policy/credibility-in-neuroscience/). Such research often leads to findings that are not reproducible, hindering the translation of discoveries into clinical applications. This not only wastes valuable resources and funding but also patient and volunteer’s time and effort, which compromises the integrity of the field and undermines trust in scientific outcomes.

Academic clinicians remain uniquely placed to bridge the gap between theoretical neuroscience and clinical practice, identify knowledge gaps and most crucially identify which research questions really matter to patients and mental health professionals. They are also perfectly positioned to train the next generation of research-active clinicians and mental health researchers.

The Royal College of Psychiatrists’ neuroscience project, funded by the Gatsby Foundation, recognised the growing complexity of the science underlying our understanding of mental health conditions. It led to major changes in psychiatric training, to allow all psychiatrists to have the skills to understand the emerging evidence in mental health neuroscience. Research is everybody’s business in mental health services and now is the time to build on what has come before, with high-quality studies, in order to make a real difference to the lives of people affected by mental illness. Academic psychiatrists have a key role to play in ensuring that studies aiming to improve understanding of mental health conditions or to develop new treatments are well performed and reproducible. Clinical services and academic institutions need to work better together to achieve this because research matters to everyone working in mental health services.
